# Cardiac function in *Vimba vimba* embryos under electromagnetic exposure at hatchery-relevant intensities

**DOI:** 10.1371/journal.pone.0334035

**Published:** 2025-10-08

**Authors:** Jan Krzystolik, Adam Tański, Camille L. Musseau

**Affiliations:** 1 Department of Hydrobiology, Ichthyology and Biotechnology of Animal Reproduction, West Pomeranian University of Technology in Szczecin, Szczecin, Poland; 2 Department of Fish Biology, Fisheries and Aquaculture, Leibniz Institute of Freshwater Ecology and Inland Fisheries, Berlin, Germany; inSync Mirror LLC, UNITED STATES OF AMERICA

## Abstract

The increasing expansion of energy infrastructure and anthropogenic transformation of the environment have introduced electromagnetic fields (EMFs) into aquatic ecosystems. While studies on the impact of EMFs on aquatic organisms are growing, their effects on fish embryonic development remain poorly understood. This study investigated the influence of electromagnetic exposure dominated by the magnetic (B) component, measured at 11.15 (± 2.24) μT and 50 Hz, on the heart rate of vimba bream (*Vimba vimba*) embryos, a species of conservation significance in aquaculture. The electric (E) component was not measured, and its potential contribution cannot be excluded. Fertilized *V. vimba* eggs were incubated under control conditions or exposed to an EMF, and embryonic heart rate was monitored in two experimental series to assess both short- and long-term EMF effects. In Experiment 1, embryos were incubated either under EMF (Variant B, *n* = 18) or control conditions (Variant A, *n* = 18) and observed without additional exposure. In Experiment 2, embryos incubated under EMF (Variant D, *n* = 30) or control conditions (Variant C, *n* = 30) were exposed to EMF during microscopic observation. Bayesian non-linear mixed models revealed significant EMF effects in both experiments. Embryos exposed to EMF during incubation (Variant B) displayed a 23.5% higher baseline heart rate (95% CI: 14.5–31.9%) compared to controls. In Experiment 2, embryos exposed to EMF during observation (Variant D) showed a rapid heart rate increase of 29.0% (95% CI: 21.5–37.6%). Embryos exposed to EMF during incubation exhibited a diminished response to subsequent EMF exposure, suggesting physiological adaptation. Additionally, EMF exposure during incubation was associated with reduced inter-individual variability in heart rate, suggesting a homogenizing effect on embryonic cardiac responses. These findings demonstrate measurable cardiac responses of fish embryos to hatchery-relevant electromagnetic exposure and highlight compensatory mechanisms regulating heart rate. While the results suggest fish embryos can adapt to such electromagnetic exposure, further studies are required to disentangle the roles of both magnetic and electric components and to evaluate long-term physiological and ecological consequences.

## Introduction

Natural geomagnetic field (GMF) varies in intensity from 30 to 70 μT depending on proximity to the magnetic poles [1]. Many fish species can detect and respond to GMF [2–6]. However, due to increasing anthropogenic activity and the expansion of energy infrastructure, aquatic environments are now also exposed to electromagnetic fields (EMFs) of anthropogenic origin. Major sources of EMFs that may impact aquatic ecosystems include submarine power transmission cables, wind farms, high-voltage power lines, and power plants [7–13].

The influence of EMFs on fish is not limited to natural environments, as these fields are also present in aquaculture facilities. The highest EMF levels are expected in recirculating aquaculture systems (RAS), where numerous electrical devices are in operation. In such systems, magnetic fields may even be used to modify water parameters, including increasing dissolved oxygen content, pH, and electrical conductivity, while reducing ammonia and ammonium concentration, which could directly or indirectly affect fish or their developing eggs [14–16]. It has already been demonstrated that electrical devices can influence certain aspects of egg incubation, such as the directional orientation of embryos and their survival rate [17]. Despite ongoing research, the effects of EMFs on fish remain poorly understood, with studies often yielding inconsistent results. In natural conditions, some migratory fish species have been observed to alter their migration routes in response to EMFs, either individually or at the population level [8,18–20]. However, while there are indications that EMFs may influence fish migration, their overall impact on adult fish is still considered minor or insufficiently documented to draw definitive conclusions [11,21]. Observations of larval behavior under EMF exposure also provide conflicting results. Even studies conducted under identical conditions have reported EMF-induced effects in some species [22], while others remained unaffected [23].

This study focuses on the impact of EMFs on fish embryonic development. Research on zebrafish (*Danio rerio*) embryos has shown that EMFs can slow growth, increase oxidative stress gene transcription, trigger apoptotic processes, and alter cholesterol metabolism [24]. Studies on developing roach (*Rutilus rutilus*) eggs indicate that EMFs can accelerate hatching time, increase morphological variability in larvae, reduce body length and weight, alter vertebral counts in juveniles, and even increase embryonic and larval mortality [25,26]. While these findings underscore the potential for EMFs to disrupt early fish development, the physiological mechanisms underlying these effects remain poorly characterized. One particularly critical and sensitive endpoint is embryonic cardiac function, which can serve as an early biomarker of stress, toxicity, or developmental disruption [27–31]. Heart rate alterations may reflect systemic effects on metabolism, ion channel function, or neuroendocrine signaling pathways during organogenesis [26,32–34]. Additionally, there is evidence that EMFs may affect heart activity in fish embryos during organogenesis. Research on static magnetic fields (SMFs) suggests they can influence heart function in developing fish embryos [35,36]. However, SMFs differ from EMFs in their mechanisms of action and sources [37,38]. Moreover, no studies to our knowledge have directly investigated how anthropogenic EMFs, particularly those at 50/60 Hz typical of power infrastructure, affect heart function in fish embryos during early development. This represents a significant gap in understanding, especially given the ubiquity of EMF sources in freshwater aquaculture facilities, riverine habitats near power lines, and hatchery settings. Therefore, this study specifically examines the effects of 50 Hz EMFs on cardiac function, focusing on heart rate as a physiological endpoint, we aim to provide mechanistic insight into EMF-induced stress during early vertebrate development and to inform potential environmental risk assessments related to aquatic EMF exposure. Low-frequency electromagnetic fields, such as 50 Hz, consist of two components: an electric field (E) and a magnetic field (B). In aquatic environments, the magnetic field penetrates water freely, while the electric field is significantly attenuated due to water’s high conductivity [39,40].

The vimba bream (*Vimba vimba*) was selected as the model species for this study. This rheophilic fish from the family Leuciscidae is capable of anadromous migrations. Once an important target for fisheries, *V. vimba* populations have declined significantly in many of their former habitats due to environmental degradation and overfishing [41–45]. The growing need for conservation efforts has led to the increasing use of this species in conservation aquaculture programs, where individuals are bred for restocking purposes [46–48]. The choice of *V. vimba* for this study was driven by the need to assess whether anthropogenic EMFs could influence its embryogenesis, specifically by affecting heart activity in developing embryos. Understanding these effects could provide valuable insights into the potential risks or adaptive responses of *V. vimba* populations to EMF exposure, both in natural spawning grounds and in conservation aquaculture facilities.

In this study, we aimed to quantify the effects of EMF on vimba bream embryos through two separate experiments. The first investigated the impact of EMF exposure on embryos after the eggs had been exposed during incubation. The second examined the effects of EMF exposure during both egg incubation and embryo observation. Based on previous research indicating that fish can detect and respond to electromagnetic fields, and given evidence of EMF-induced physiological changes in other fish species, we hypothesize that exposure to anthropogenic EMFs will influence the heart activity of *V. vimba* embryos during early development. Based on known physiological stress responses in fish [49], we expected that EMF exposure would initially increase embryonic heart rate.

## Materials and methods

### Materials

The study was conducted on fertilized *Vimba vimba* eggs obtained in collaboration with the Polish Angling Association, Szczecin District (ul. Mickiewicza 3, 70–383 Szczecin, Poland). Broodstock were collected using electrofishing from a boat on the Ina River near Goleniów (53°33’54’‘ N, 14°47’50’‘). Before gamete collection, the captured fish were first subjected to thermal stimulation by gradually increasing the water temperature by 2°C over four days. This was followed by hormonal stimulation with two doses of Ovopel [50,51]. The fish, consisting of 11 females and 6 males, were acclimated in aerated 1,000 L tanks before the procedure. Gametes were obtained through hand stripping, after which the broodstock were released back into the Ina River. Approximately 425,000 eggs were collected in total. Following fertilization, the eggs were treated to prevent adhesion. For the experiment, 2,400 eggs were randomly selected from the available batch, while surplus eggs were sent for incubation at the Hatchery and Stocking Center in Goleniów. The resulting fry were reared and later released into inland waters.

The fertilized eggs were transported to an isothermal laboratory, where a constant temperature was maintained throughout the experiment. The eggs were volumetrically divided [52,53] into eight batches of approximately 300 eggs each. Each batch was incubated in a separate tank with internal dimensions of 30 cm × 30 cm × 30 cm, with controlled water circulation and aeration at 18°C, ensuring optimal developmental conditions [54,55]. Water temperature and dissolved oxygen was measured daily and remained stable throughout the experiment, ranging from 17.9°C to 18.2°C across all tanks, dissolved oxygen concentration remained between 9.36 mg/L and 9.59 mg/L. Water pH was measured at the beginning and end of the incubation period and remained stable at 7.4, consistent with values typical of local freshwater used in hatchery settings. Four tanks served as controls, where eggs were incubated without EMF exposure, while the remaining four tanks were exposed to EMF. To simulate electromagnetic conditions commonly found in household and aquaculture facilities, an air compressor from a refrigeration unit was used as the EMF source. To minimize potential vibrations that could influence embryonic behavior, the compressor was placed on a specially designed anti-vibration platform constructed from rubber and foam. The EMF source could generate both electric (E) and magnetic (B) components. In this experiment, only the magnetic component (B field) was measured, and all results are expressed in terms of magnetic flux density. Due to the design of the setup, including insulation of wiring and lack of electrodes in water, the direct contribution of the electric component was expected to be strongly attenuated [39,40,56]. However, since no direct measurements of the electric field were performed, its potential contribution cannot be excluded. The study therefore focused on realistic exposure dominated by the magnetic field component, while explicitly acknowledging that the role of residual electric fields could not be established within this experimental design

In the applied experimental setup, the intensity of the EMF acting on the eggs could only be regulated by adjusting the distance between the incubation tank and the EMF source. To determine an appropriate and ecologically relevant magnetic flux density (B field) for the experiment, field measurements were conducted in a functioning aquaculture facility—specifically, in the hatchery of the Polish Angling Association, Szczecin District (53°33′9.786677″N, 14°50′26.401991″E), which specializes in the production of early developmental stages of fish for restocking programs. Magnetic flux densitywas recorded at ten points surrounding the incubation systems. Measured values ranged from 0.31 μT to 22.24 μT, depending on the proximity to operational electrical equipment.

Based on these field observations, the experimental setup aimed to replicate average values commonly observed in aquaculture facilities, targeting a magnetic flux density of approximately 11 μT.

Magnetic field measurements were performed along a horizontal line at the bottom of each tank, at the level where the embryos were located. The field intensity was measured in 10 equidistant points (every 3 cm), starting from the tank wall closest to the EMF source to the opposite wall along the central longitudinal axis of the tank. All measurements were taken using a three-axis waterproof EMF meter (3-Axis Electromagnetic Field Meter TM-192Det), oriented with the sensor directed towards the EMF source. The device measures the root mean square (RMS) value of the magnetic flux density (µT) along three orthogonal axes. The aim of the measurements was not to obtain a complete three-dimensional mapping of the field, but rather to confirm realistic exposure conditions representative of aquaculture systems, where electrical equipment is commonly used. Initially, the first tank was positioned such that the magnetic flux densitymeasured 14.56 at the first measurement point, located closest to the EMF source. The remaining three tanks were subsequently arranged symmetrically around the source, and their placement was adjusted so that, at each of the ten corresponding measurement points, the magnetic flux density deviated by no more than ±0.2 μT from the values recorded at the same positions in the reference tank. As a result, the magnetic flux density values recorded across all four EMF-exposed tanks ranged from 8.21 to 14.81 μT, with a mean of 11.15 ± 2.24 μT. The primary frequency component of the field was 50 Hz. Minor contributions from higher harmonics (notably 100 and 150 Hz) were also detected, but their influence on the overall magnetic field intensity was negligible. It should be noted that the tanks containing incubating eggs are located in the near field of the electromagnetic source, where significant spatial variability of the field can be expected due to the irregular geometry of the source. Therefore, the measurements conducted according to the presented methodology—limited to the longitudinal axis of the tank (X)—and excluding measurements along the width (Y) and height (Z) axes, should be considered indicative. The electromagnetic field values affecting the incubating eggs along the other axes may differ; however, as demonstrated by analyses carried out on the experimental setup, they do not exceed a few microteslas (μT). To ensure random spatial distribution of embryos within each incubation tank, each subsample of 300 eggs was gently poured into the tank, after which the water was briefly stirred to promote even dispersal of the eggs across the bottom. Exposure to the electromagnetic field began approximately 35 minutes after fertilization, when the embryos were placed into the incubation tanks at the early cleavage stage, following the initial cell divisions. The exposure continued uninterrupted throughout the incubation period until the embryos reached advanced organogenesis, at which point heart rate measurements were conducted. Embryonic development was monitored throughout the entire incubation period. Once the embryos reached the organogenesis stage and heart activity became observable, the next phase of the experiment was initiated. Observations were conducted using microscopes (Nikon 2000SE, Tokyo, Japan) equipped with cameras (Nikon Digital Sight 10, Tokyo, Japan), and heart rate measurements were obtained from recorded videos. The image was transmitted directly to a computer screen, and heartbeats were manually counted by observing the rhythmic contractions in slow-motion playback. Videos were recorded at a frame rate of 60 frames per second (fps) with a resolution of 1920 × 1080 pixels using the Nikon Digital Sight 10 camera. To ensure random sampling, embryos for observation were collected sequentially from each of the four incubation tanks, beginning with tank 1, followed by tanks 2, 3, and 4, then repeating the sequence. Within each tank, individual embryos were selected randomly without regard to their position relative to the EMF source. The same sampling protocol was applied to both the EMF-exposed and control groups.

### Experimental set up

#### Experiment 1.

**Fig 1:**
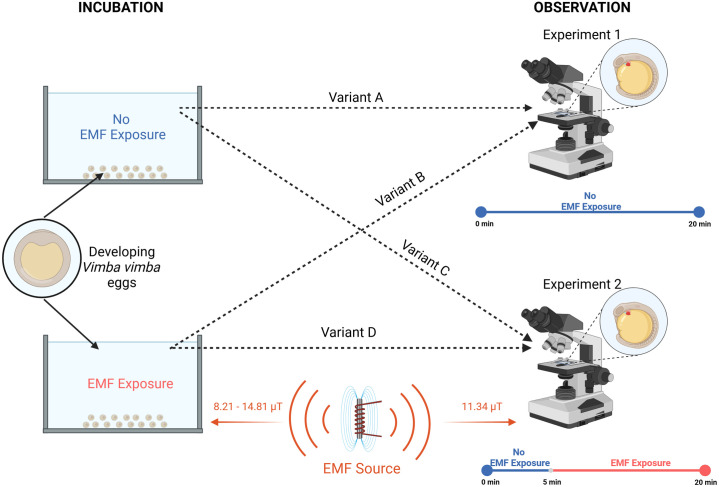
Diagram of the experimental setup.

In the first experiment (Fig 1), embryos were observed under a microscope for 20 minutes to compare heart rates between those incubated with and without EMF exposure. Heart rate was recorded at 1, 3, 5, 6, 8, 10, 15, and 20 minutes. Each of the following experimental variants was replicated on 18 individuals:

- Variant A – Observation of V. vimba eggs incubated without EMF exposure, with no EMF applied during observation.
- Variant B – Observation of V. vimba eggs incubated under EMF exposure, with no EMF applied during observation.

#### Experiment 2.

The second experiment followed (Fig 1) the same methodology as the first one but included the introduction of EMF exposure at the fifth minute of observation. The EMF was generated by the same compressor used during incubation. The compressor was positioned to ensure that the observed embryos were exposed to a magnetic flux density of 11.34 (± 0.31) μT at 50 Hz. This value was confirmed through three repeated measurements with the EMF meter placed exactly at the location of the observation dish under the microscope.

Each of the following experimental variants was replicated replicated on 30 individuals

- Variant C – Observation of *V. vimba* eggs incubated without EMF exposure, with EMF introduced at the fifth minute of observation.
- Variant D – Observation of *V. vimba* eggs incubated under EMF exposure, with EMF introduced at the fifth minute of observation.

All studies were carried out in accordance with the European directive (2010/63/EU) and Polish regulations concerning experiments on animals, as per the Act (2015/266). For the described procedures, there was no need for approval by the Ethics Committee, as the eggs and larvae, until the completion of the yolk-sac absorption, did not qualify as animals, which excluded them from the scope of the directive. For the described procedures, no approval from the Ethics Committee was required, as the eggs and larvae of lower vertebrates, until the completion of yolk-sac absorption, are not classified as animals for which ethical approval is necessary.

### Statistical analyses

The effects of EMF exposure on embryonic heart activity in Experiments 1 and 2 were assessed using Bayesian generalized additive mixed models (GAMMs), which extend linear mixed-effects models by allowing flexible, non-linear relationships between predictors and the response variable. These models account for repeated measurements within individuals, as heart rate was recorded multiple times on the same individuals, leading to non-independent observations [57]. Furthermore, individual embryos may exhibit intrinsic differences in baseline heart rate due to biological variability and not explained by variables of interest tested in the models. By incorporating random intercepts, GAMMs allow each individual to have its own baseline heart rate while still estimating overall treatment effects, thereby capturing inter-individual variability unexplained by fixed effects. In the present models, fixed effects estimated differences in baseline heart rate at t = 0 between variants, as well as the average temporal change in heart rate over the full 0–20 min observation period for each variant, obtained from the fitted smooth terms. Variant-specific smooth terms for time were included to capture non-linear changes in heart rate trajectories, while the variability in these smooths (“spline smooth variability”) quantified how much individual time courses deviated from the average curve within each variant. Random intercept variability represented between-individual differences in baseline heart rate. For comparisons between groups, we calculated the difference in the average temporal change (Δ) as the difference between the slopes of the smooth terms for each variant, and expressed it in both absolute terms (bpm) and relative terms as a percentage of the slope in the reference variant.

Two separate generalized additive mixed models (GAMMs) were constructed to assess the effects of EMF exposure on embryonic heart rate over a 20-minute observation period.

For Experiment 1 (Variant A: non-exposed; Variant B: exposed during observation), the GAMM included a fixed effect of exposure group to test for differences in baseline heart rate at the start of the experiment (time = 0), and group-specific smooth terms for time to capture potential non-linear changes in heart rate trajectories within each variant.

For Experiment 2 (Variant C: non-exposed; Variant D: exposed during incubation), a separate GAMM was specified with fixed effects of incubation group and EMF exposure during observation, as well as their interaction, to evaluate whether baseline heart rate and responses to EMF differed between groups. Group-specific smooth terms for time were again included to model dynamic changes in heart rate over time.

In both models, individual embryos were included as random intercepts to account for repeated measurements and intrinsic variability in baseline heart rate.

The models were fitted using the R package ‘brms’ [58], which implements Bayesian inference via Stan [59]. Each model was run with four Markov chains, 4,000 iterations per chain, including a 2,000-iterations warm-up and a control stetting of adapt_delta = 0.99 to improve convergence. Posterior convergence was assessed by visual inspection of trace plots and by ensuring that all Ȓ (Gelman-Rubin diagnostic) values were < 1.01.

All statistical analyses were performed using RStudio (2024.09.1), and figures were generated using the R package ggplot2 [60].

## Results

### Experiment 1

**Fig 2:**
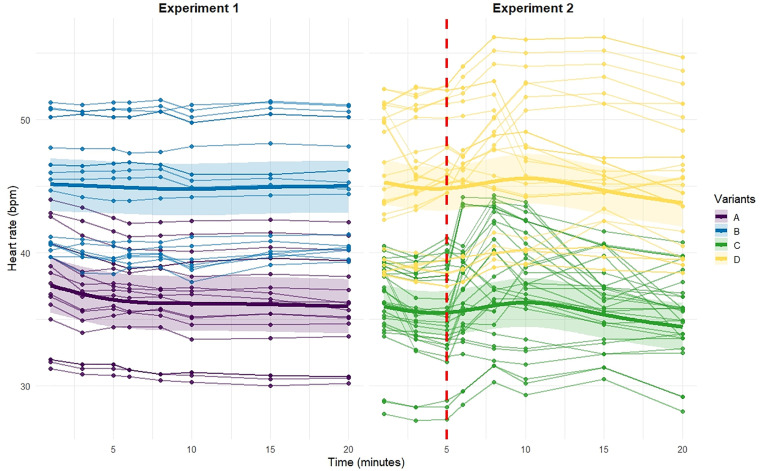
Heart rate (bpm) response over time (0 to 20 minutes) for individual embryos (thin lines) and the marginal posterior means (thick lines) with associated 95% credible intervals (shaded areas) obtained from Bayesian GAMMs for each variant. Experiment 1 (left panel) shows Variant A (purple) and Variant B (blue), while Experiment 2 (right panel) displays Variant C (green) and Variant D (yellow). The vertical red line in the right panel indicates the start of EMF exposure at 5 minutes.

**Fig 3:**
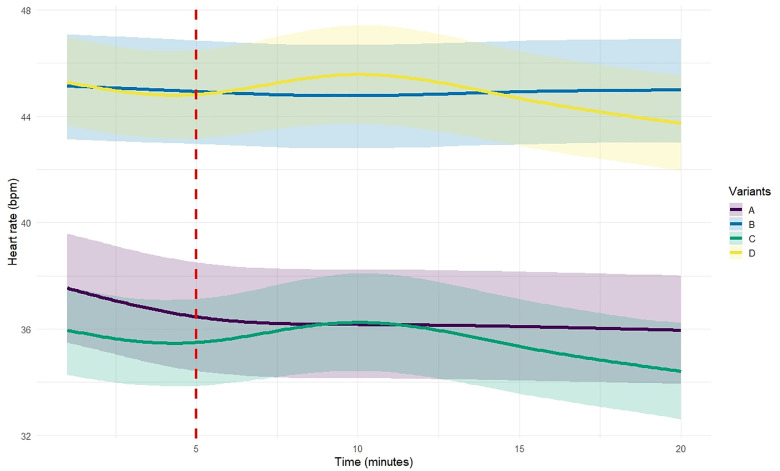
Heart rate (bpm) response over time (0 to 20 minutes) for each variant (Experiment 1: Variants A and B; Experiment 2: Variants C and D), as estimated by Bayesian GAMMs. Solid lines represent the marginal posterior means, and shaded areas indicate the 95% credible intervals. The vertical red line in the right panel marks the onset of EMF exposure at 5 minutes in variants C and D.

Exposure to EMF during incubation led to an elevated baseline heart rate. At t = 0, embryos in Variant A (non-exposed) had a baseline heart rate of 36.43 ± 1.01 bpm (95% CI: 34.45–38.43 bpm). In contrast, embryos in Variant B (exposed to EMF during incubation) showed a 23.5% higher baseline heart rate (95% CI: 15.1% – 31.9%), with an average of 44.96 ± 1.75 bpm (95% CI: 41.52–48.40 bpm) (Figs 2 and 3, S1 Table).

Heart rate dynamics over time displayed non-linear patterns that differed markedly between groups. In Variant A, heart rate decreased significantly over time (posterior mean: −6.85, 95% CI: −8.45 – −5.27), while in Variant B, no significant temporal trend was detected (posterior mean: −0.80, 95% CI: −2.23–0.55). The difference in temporal trajectories between groups was itself significant (Δ = +6.04 bpm, 95% CI: + 3.0 – + 8.7 bpm), indicating that prior EMF exposure attenuated the time-dependent decline in heart rate observed in non-exposed embryos. This attenuation corresponds to an 88% reduction in temporal change (95% CI: 60% – 116%), suggesting that EMF exposure during incubation nearly suppressed the decline over time seen in Variant A (Figs 2 and 3, S1 Table).

Random effects indicated considerable variability in baseline heart rate between embryos (sd = 4.31 bpm, 95% CI: 3.41–5.46). Temporal trajectories were more variable in Variant A (sd = 3.40 bpm, 95% CI: 1.25–8.33) than in Variant B (sd = 1.46 bpm, 95% CI: 0.32–4.79), suggesting that prior EMF exposure may homogenize the temporal heart rate response across individuals (S1 Table).

### Experiment 2

The effects of EMF exposure during incubation, time and EMF exposure during observation, were statistically significant (Figs 2, and 3 and S2 Table). Exposure to EMF during incubation led to an increase in baseline heart rate. The baseline heart rate for Variant C (non-exposed embryos) was estimated at 34.83 ± 0.77 bpm (95% CI: 33.30–36.32). Embryos in Variant D (exposed during incubation) exhibited a significantly higher baseline heart rate, with an increase of 10.11 ± 1.19 bpm (95% CI: 7.80–12.51), corresponding to a 29% (95% CI: 21.5–37.6) higher resting heart rate compared to Variant C.

Heart rate decreased over time in a non-linear manner, as captured by group-specific smooth terms in the model. In Variant C, this temporal decline was clearly pronounced, with an overall estimated decrease of −8.82 bpm (95% CI: −15.32 to −4.14). In Variant D, the decline was less marked, with an estimated effect of −3.48 bpm (95% CI: −9.99 to +1.75); however, the credible interval included zero, indicating greater uncertainty about this effect. Compared to Variant C, the reduction in heart rate over time in Variant D was approximately 61% smaller (95% CI: −14% to 188%), reflecting an attenuated but uncertain temporal decline in this group.

Additional exposure to EMF after minute 5 differentially affected the two variants. In Variant C, heart rate increased by 2.54 bpm (95% CI: 1.72–3.34), corresponding to a 7.3% increase relative to the baseline (95% CI: 4.7–10.0). In Variant D, this EMF-induced increase was attenuated by −1.57 bpm (95% CI: −2.77 to −0.40). Overall, although both variants showed an increase in heart rate following EMF exposure, the effect in Variant D was approximately 62% smaller than in Variant C (95% CI: 36% – 77% reduction), indicating a substantially reduced responsiveness to EMF after prior exposure during incubation (Figs 2 and 3, S2 Table).

Random effects captured inter-individual differences in baseline heart rates and temporal dynamics. The standard deviation of the random intercepts was 4.52 bpm (95% CI: 3.61–5.71), reflecting variability in baseline heart rates across embryos. The temporal trajectories showed more variability in Variant C, with a random slope standard deviation of 4.21 bpm (95% CI: 1.50–9.15), than in Variant D (2.87 bpm, 95% CI: 0.71–7.48), suggesting that embryos previously exposed to EMF exhibited more homogeneous temporal patterns (S2 Table).

## Discussion

The results of this study demonstrate a significant impact of EMFs on heart rate in *Vimba vimba* embryos. Two types of EMF exposure were distinguished based on duration: long-term exposure, from fertilization until the start of observations (variants B and D), and short-term exposure, from the 5th to the 20th minute of microscopic observation (variants C and D). In both cases, EMF exposure led to an increase in heart rate. Experiment 1 revealed that exposure of the eggs to EMF during incubation resulted in an average 23.5% increase in heart rate compared to eggs incubated only in GMF. This increase, representing a substantial elevation in cardiac activity, may have significant physiological implications for embryonic development, as elevated heart rates often correlate with increased metabolic demand and potential developmental stress. The observed 88% attenuation of the normal temporal decline in heart rate further suggests a disruption of typical developmental heart rate plasticity, possibly indicating altered cardiac regulatory mechanisms. Studies on other fish species have shown that elevated embryonic heart rates, can correlate with earlier hatching, smaller larval size, or reduced survival post-hatching due to energetic trade-offs [61,62]. In Experiment 2, short-term exposure to EMF during observation caused a rapid heart increase, with variant C showing a 2.54 bpm acceleration shortly after exposure, corresponding to a 7.3% increase relative to the baseline.

Few studies have examined the effects of EMFs on heart rate in fish at early developmental stages. Krylov et al. [63] reported an accelerated heart rate in zebrafish (*Danio rerio*) embryos exposed to EMFs, particularly when exposure began at early ontogenetic stages. Similarly, increased heart rate was observed in sea trout (*Salmo trutta*) and European whitefish (*Coregonus lavaretus*) subjected to high-intensity EMFs (15 mT, 50 Hz) [64]. In European whitefish, heart rate acceleration occurred within the first minute of EMF exposure, mirroring the findings of this study. However, in sea trout, a pronounced response emerged only after three minutes of exposure. Unlike the present study, neither of these experiments reported the subsequent stabilization of heart rate and its return to baseline over time. Interestingly, static magnetic fields (SMFs) of 4 mT induced a nearly identical acceleration of heart rate in the same fish species [36,64]. SMFs with intensities ranging from 1 to 5 mT also caused an acceleration of heart rate in aquarium-reared fish *Parachromis managuensis* and *Andinoacara rivulatus* [65]. At much higher SMF intensities (51–70 mT), Formicki and Winnicki [35] also observed heart rate acceleration in common carp (*Cyprinus carpio*). Notably, studies on European whitefish and sea trout exposed to SMFs revealed a response pattern similar to that observed in this study: an initial increase in heart rate, followed by a gradual decline and return to baseline values. These findings suggest a consistent trend of heart rate acceleration in fish embryos exposed to EMFs.

Research on other vertebrates also supports the hypothesis that EMFs increase heart rate. For instance, studies on chicken (*Gallus gallus domesticus*) embryos demonstrated that long-term exposure to EMFs at 50 Hz, with intensities of 50 and 100 μT, increased heart rate [66]. Similarly, adult rats exposed to relatively strong SMFs (0.7 T) exhibited significant heart rate acceleration [67]. However, in studies on the same species subjected to short-term EMF exposure at 1 μT, 50 Hz, no significant effect on heart rate was detected [68]. In humans, increased heart rate and arrhythmia were reported among individuals exposed to EMFs generated by electric trains [69]. However, other studies [70–72] did not confirm any EMF-induced alterations in human heart rate. This brief review of studies on vertebrates other than fish suggests that the effects of magnetic fields are not as consistent as those observed in fish embryos. Nevertheless, in many cases, EMF exposure has been associated with heart rate acceleration.

The exact mechanisms underlying EMF-induced changes in heart rate remain unclear. Most studies focus on documenting EMF effects rather than identifying the physiological processes responsible for these changes. Given the limited research on fish, insights into possible mechanisms must be drawn from studies on other vertebrates.

Several physiological mechanisms have been proposed to explain EMF-induced heart rate acceleration. One possibility is the disruption of autonomic nervous system (ANS) balance, leading to sympathetic dominance and parasympathetic suppression. Such changes are characteristic of the “fight-or-flight” response and result in increased heart rate [72–74]. Since EMF exposure has been associated with both increased heart rate and irregular cardiac rhythms, this suggests a potential impact on central cardiovascular regulation [75]. Another possible mechanism is hypocalcemia, observed in rats subjected to prolonged EMF exposure. Reduced blood calcium levels may affect cardiac electrical conduction, increasing cardiomyocyte excitability and accelerating heart rate [76–78]. Hypocalcemia has also been linked to prolonged P-R intervals in rodents, indicating impaired atrioventricular node conduction [76,79].

EMFs may also enhance the activity of the renin-angiotensin-aldosterone system (RAAS), leading to elevated angiotensin II levels. This molecule induces vasoconstriction and increases blood pressure, ultimately overloading the heart and accelerating its function [76,80,81]. Additionally, excessive RAAS activity has been associated with alterations in cardiac ion channels, potentially causing ventricular repolarization disturbances and QTc prolongation, which may contribute to arrhythmias and tachycardia [76,82].

Oxidative stress represents another plausible mechanism for EMF-induced heart rate acceleration. Increased production of reactive oxygen species (ROS) and a weakened antioxidant defense system can result in cellular damage. EMF exposure has been shown to reduce the activity of free radical-neutralizing enzymes such as superoxide dismutase (SOD) and glutathione peroxidase (GPX), while promoting lipid peroxidation and membrane damage [83–85]. Excessive ROS production can impair mitochondrial function and reduce the heart’s ability to produce energy efficiently, leading to a compensatory increase in heart rate [76].

Observations from Experiment 2 indicate that heart rate initially increases upon EMF exposure, reaching its peak three minutes after exposure. Following this peak, the heart rate gradually slows down and, within a relatively short time, returns to pre-exposure levels, despite the continued presence of EMF.

The negative non-linear relationship between heart rate and time suggests a potential adaptation or habituation process, in which embryos gradually adjust to the study conditions over time. This experimental study revealed that following an initial increase in heart rate – peaking three minutes after EMF exposure – the heart gradually slowed and returned to pre-exposure levels within a relatively short period, despite continued EMF presence (variants C and D). This response may reflect an adaptive compensatory mechanism, where an initial stress reaction triggers physiological processes aimed at restoring homeostasis. Jeong et al. [75] demonstrated that short-term (1-day) exposure to extremely low-frequency (ELF) electromagnetic fields increased heart rate, whereas prolonged (5-day) exposure had no significant effect. This suggests that adaptive mechanisms allow organisms to mitigate the impact of EMFs over time. Additionally, Mohamed et al. [60] found that rats exposed to EMFs for 4 and 8 weeks exhibited reduced total antioxidant capacity and elevated MDA levels (a marker of oxidative stress), indicating an attempt to counteract the long-term effects of EMF exposure. The ability to compensate may also involve autonomic nervous system (ANS) regulation. Andrzejak et al. [73] observed that in humans, EMF exposure initially increased parasympathetic activity, which subsequently stabilized heart function.

In Experiment 2, the interaction between group and time indicates that heart rate changes over time differ between embryos exposed to EMF during incubation and those that were not. While both groups exhibited a heart rate increase following the onset of EMF exposure at t = 5 min, the magnitude of the heart rate increase in Variant D following EMF exposure was 48.2% lower compared to Variant C, based on the model-estimated change in beats per minute. This pattern is clearly illustrated in Fig 3, where the increase in heart is more pronounced in Variant C than in Variant D, despite overall heart rate values in Variant C remaining lower than those in Variant D. This phenomenon may also be attributed to adaptation. Jeong et al. [75] reported that organisms subjected to prolonged EMF exposure exhibited a weaker oxidative stress response compared to those experiencing EMF exposure for the first time. Similar findings were reported by Mohamed et al. [76], who observed that long-term EMF exposure reduced the cardiovascular response to subsequent exposure, potentially due to compensatory regulation of renin activity and ion balance stabilization. Additionally, Korpinen et al. [86,87] suggested that the effects of EMFs on heart function may depend on exposure dose and frequency, with prior exposure enhancing resistance to subsequent EMF influences. Another important result from both experiments concerns the variability of responses among individual embryos. The random effects analysis revealed that embryos exposed to EMF during incubation (variants B and D) exhibited reduced inter-individual variability in baseline heart rate and temporal dynamics compared to non-exposed embryos (variants A and C). This homogenization of physiological responses may suggest that EMF exposure reduces the plasticity or flexibility of embryonic cardiac regulation, potentially limiting the ability of individuals to adjust to environmental or developmental challenges. Such decreased variability could be an indicator of stress-induced constraint on normal developmental trajectories.

The phenomenon of adaptation to EMF exposure was further examined in an experiment by Piccinetti et al. [24], which investigated the effects of EMFs on zebrafish (*Danio rerio*) embryonic development between 24 and 72 hours post-fertilization. At 48 hours post-fertilization, embryos exhibited reduced growth, increased transcription of oxidative stress-related genes, initiation of apoptotic/autophagic processes, and alterations in cholesterol metabolism. However, these embryos activated detoxification mechanisms, and by 72 hours, they had recovered to a pre-stress state, achieving hatching times comparable to the control group.

## Conclusions

This study contributes to understanding EMF effects under aquaculture-relevant conditions, showing that *Vimba vimba* embryos exposed to EMFs exhibited accelerated heart rates. This response was observed under both short-term exposure (variants C and D) and long-term exposure (variants A and B), indicating that early embryonic stages of *V. vimba* may be sensitive to electromagnetic fields..

The findings also highlight the strong adaptive capacity of *V. vimba* embryos. After an initial sharp increase in heart rate, a gradual stabilization was observed, with heart rate returning close to baseline levels in the final minutes of observation. This biphasic pattern of response, marked by acute stress followed by partial compensation, suggests dynamic regulation of cardiac function under EMF exposure, possibly involving short-term physiological adaptation. Such a mechanism may reflect broader developmental plasticity in response to environmental stressors. Furthermore, microscopic analysis revealed that embryos previously exposed to EMFs during incubation (variant D) exhibited a significantly lower increase in heart rate than those encountering EMFs for the first time (variant C). This further supports the existence of adaptive mechanisms that reduce sensitivity to repeated EMF exposure.

Nevertheless, the present study has certain limitations that should be acknowledged. First, no sham control was included, which prevents full exclusion of potential confounding factors such as noise or vibration. Second, while key water quality parameters such as temperature, pH, and oxygen saturation were monitored, we did not continuously assess a broader range of physicochemical factors throughout the incubation and observation. Another limitation of the current study is that magnetic flux density was measured only along a single horizontal axis (X) at the bottom of the tank, which provides an indicative rather than complete characterization of the spatial field distribution. Moreover, only the magnetic component (B field) was measured, and although the setup design likely attenuated the electric component, its residual contribution cannot be excluded. Future studies should aim to address these limitations by incorporating more rigorous environmental monitoring and appropriate sham controls to further isolate the specific effects of EMF exposure.

While these results indicate measurable effects of EMF exposure on the cardiovascular function of V. vimba embryos, many questions remain regarding the broader effects of anthropogenic electromagnetic fields on aquatic life. As *Vimba vimba* is both a species of conservation concern and a target of supportive aquaculture programs, these findings may help inform hatchery management practices and environmental risk assessments aimed at minimizing EMF-related developmental disruptions in early life stages. Future research should aim to explore the underlying physiological mechanisms driving the observed changes in heart rate, particularly focusing on endocrine regulation, ion channel dynamics, and metabolic responses to EMF exposure. Additionally, studies incorporating a wider range of EMF intensities and exposure durations are warranted to assess threshold levels and potential dose–response relationships. Long-term effects of early EMF exposure on larval survival, behavior, and fitness should also be examined, particularly in the context of hatchery design and regulatory standards for aquaculture environments.

## Supporting information

S1 Table Summary of Bayesian Generalized Additive Mixed Model (GAMM) results for Experiment 1. Fixed effects represent the estimated variant-level effects of variant membership and time on heart rate. “Spline smooth variability” corresponds to the estimated variability in the smooth time trajectories across individuals within each variant (A and B). “Random intercept variability” reflects between-subject variability in baseline heart rate. Estimates are posterior means, with associated standard errors and 95% credible intervals (CI). Ȓ values indicate model convergence (values close to 1.0 denote satisfactory convergence).
(DOCX)

S2 Table Summary of Bayesian Generalized Additive Mixed Model (GAMM) results for Experiment 2. Fixed effects represent the estimated variant-level effects of variant membership and time on heart rate. “Spline smooth variability” corresponds to the estimated variability in the smooth time trajectories across individuals within each variant (C and D). “Random intercept variability” reflects between-subject variability in baseline heart rate. Estimates are posterior means, with associated standard errors and 95% credible intervals (CI). Ȓ values indicate model convergence (values close to 1.0 denote satisfactory convergence).
(DOCX)

## Abbreviations

EMF: electromagnetic field
GMF: geomagnetic field
SMF: static magnetic field
ANS: autonomic nervous system
RAAS: renin-angiotensin-aldosterone system
ROS: reactive oxygen species
SOD: superoxide dismutase
GPX: glutathione peroxidase
ELF: extremely low-frequency
