# Supplementary material for: Cardiac function in Vimba vimba embryos under electromagnetic exposure at hatchery-relevant intensities
Source: PLoS One. 2025 Oct 8;20(10):e0334035. doi: 10.1371/journal.pone.0334035 (PMC12507192; doi:10.1371/journal.pone.0334035)
Supplement: S1 Table — Fixed effects represent the estimated variant-level effects of variant membership and time on heart rate. “Spline smooth variability” corresponds to the estimated variability in the smooth time trajectories across individuals within each variant (A and B). “Random intercept variability” reflects between-subject variability in baseline heart rate. Estimates are posterior means, with associated standard errors and 95% credible intervals (CI). Ȓ values indicate model convergence (values close to 1.0 denote satisfactory convergence). (DOCX) [file pone.0334035.s001.docx]

## ****Table S1. Summary of Bayesian Generalized Additive Mixed Model (GAMM) results for Experiment 1.**** Fixed effects represent the estimated variant-level effects of variant membership and time on heart rate. “Spline smooth variability” corresponds to the estimated variability in the smooth time trajectories across individuals within each variant (A and B). “Random intercept variability” reflects between-subject variability in baseline heart rate. Estimates are posterior means, with associated standard errors and 95% credible intervals (CI). Ȓ values indicate model convergence (values close to 1.0 denote satisfactory convergence).

| **Parameter** | **Estimate** | **Est. Error** | **Lower 95% CI** | **Upper 95% CI** | **Ȓ** |
| --- | --- | --- | --- | --- | --- |
| *Fixed effects* |  |  |  |  |  |
| Intercept | 36.48 | 1.01 | 34.51 | 38.49 | 1.0 |
| VariantB | 8.47 | 1.41 | 5.72 | 11.31 | 1.0 |
| stime:VariantA_1 | -6.83 | 0.81 | -8.38 | -5.22 | 1.0 |
| stime:VariantB_1 | -0.79 | 0.72 | -2.27 | 0.58 | 1.0 |
| *Spline smooth variability* |  |  |  |  |  |
| sds(stimeVariantA_1) | 3.4 | 1.94 | 1.25 | 8.33 | 1.0 |
| sds(stimeVariantB_1) | 1.46 | 1.23 | 0.32 | 4.79 | 1.0 |
| *Random intercept variability* |  |  |  |  |  |
| sd(Intercept) | 4.31 | 0.53 | 3.41 | 5.46 | 1.0 |
